# Late-Onset Myocarditis Following Immune Checkpoint Inhibitors Therapy: A Case Series with Literature Review

**DOI:** 10.3390/medicina61020302

**Published:** 2025-02-09

**Authors:** Harun Muğlu, Erdem Sünger, Bahadır Köylü, Didem Tunalı, Cengiz Erol, Fatih Selcukbiricik, Ahmet Bilici, Omer Fatih Olmez

**Affiliations:** 1 Department of Medical Oncology, Faculty of Medicine, Medipol University, Istanbul 34214, Turkey; erdemsunger@gmail.com (E.S.); ahmetknower@yahoo.com (A.B.); omerfatih.olmez@medipol.com.tr (O.F.O.); 2 Department of Medical Oncology, Istanbul Koc University, Istanbul 34450, Turkey; bahadirkoylu@hotmail.com (B.K.); dtunali@kuh.ku.edu.tr (D.T.);; 3 Department of Radiology, Faculty of Medicine, Medipol University, Istanbul 34214, Turkey; cerol@medipol.edu.tr

**Keywords:** immune checkpoint inhibitors, immune-related adverse events (irAEs), late-onset myocarditis, cardiotoxicity

## Abstract

Immune checkpoint inhibitors (ICIs) therapy has revolutionized cancer treatment. However, it is important to acknowledge that ICI therapy can lead to immune-related adverse events (irAEs), including myocarditis. While early-onset myocarditis is well-documented, late-onset cases are increasingly recognized. This case series presents four cases of late-onset ICI-associated myocarditis, emphasizing the need for long-term surveillance of this potentially fatal complication. Patients exhibited a range of cardiac symptoms, including chest pain, shortness of breath, and arrhythmias. The diagnosis was confirmed through cardiac magnetic resonance imaging (MRI) and elevated cardiac biomarkers. Treatment involved the immediate discontinuation of ICI therapy and the initiation of high-dose corticosteroids. In cases with an inadequate response, additional immunosuppressive agents were considered. This case series underscores the importance of prolonged monitoring for late-onset ICI-associated myocarditis. Further research is needed to establish optimal treatment strategies and long-term management approaches for this complex condition.

## 1. Introduction

It is fair to say that immune checkpoint inhibitors (ICIs) therapy has significantly transformed cancer treatment. ICIs are monoclonal antibodies designed to target regulatory receptors on immune cells, such as programmed cell death receptor 1 (PD-1), programmed cell death ligand 1 (PD-L1), and cytotoxic T-lymphocyte-associated protein 4 (CTLA-4). By binding to these inhibitory receptors, ICIs enhance the immune system’s ability to recognize and attack cancer cells. Growing evidence suggests that ICIs have potential in the treatment of a wide range of cancers [1]. However, ICI therapy is also associated with immune-related adverse events (irAEs), including the rare but serious complication of ICI-induced myocarditis, which carries a high mortality risk (1). While ICI-induced myocarditis typically presents early in the course of immunotherapy, further investigation of delayed-onset cases (>90 days) is warranted, as they have been linked to a higher risk of major adverse cardiac events [2].

Recent evidence suggests that ICI therapy can lead to late-onset irAEs, which are frequently underreported in clinical trials. A real-world study indicated that approximately 35.2% of irAEs persist beyond six months, with nearly 40% of patients continuing to experience these effects at follow-up [2]. Additionally, FDA and EMA registration trials have often failed to document the duration of irAEs and the proportion of patients with ongoing toxicity at study completion, leading to significant gaps in long-term safety assessments [2]. Given the increasing recognition of late-onset myocarditis as a potential consequence of ICI therapy, comprehensive post-treatment surveillance strategies are essential to monitor patients for persistent toxicities and optimize long-term management [2].

Furthermore, recent reports suggest that late-onset ICI-induced myocarditis, which manifests more than 90 days after therapy initiation, remains an underrecognized clinical entity with significant implications for patient outcomes. A recent case report described a fulminant case of ICI-induced myocarditis emerging in the later stages of pembrolizumab therapy, representing one of the latest reported occurrences in the literature [3]. Notably, delayed irAEs, including cardiac complications, have been linked to an increased risk of left ventricular dysfunction and heart failure, highlighting the importance of long-term cardiac surveillance even after the discontinuation of ICIs. As the use of immunotherapy continues to expand, the early recognition and management of late-onset ICI-induced myocarditis will be crucial for optimizing patient care [3].

Despite the increasing prevalence of ICI-induced myocarditis, there is currently a lack of robust evidence to guide optimal immunosuppressive therapy. Current guidelines are primarily based on expert consensus, and randomized controlled trials evaluating the efficacy of different immunosuppressive strategies are lacking [4,5].

Recent studies suggest that immune-mediated cardiac toxicity extends beyond myocarditis and encompasses a range of adverse effects, including arrhythmias, myocardial fibrosis, and vascular inflammation. These conditions may arise due to shared immunopathological mechanisms between tumors and the heart. Autoimmune responses targeting cardiac-specific antigens, along with inflammatory remodeling, have been implicated in this process [6]. Similar patterns have been observed in other immune-driven cardiac pathologies, such as HIV-associated atrial fibrillation, where chronic immune activation and myocardial fibrosis contribute to long-term electrophysiological disturbances. Notably, studies on HIV-positive patients undergoing catheter ablation have demonstrated a significantly higher prevalence of non-pulmonary vein (non-PV) arrhythmic triggers, suggesting a distinct immune-related myocardial remodeling process. Given that ICI therapy can sustain immune activation even after treatment cessation, similar mechanisms may underlie the delayed cardiac effects observed in late-onset myocarditis [6].

Furthermore, the role of subclinical inflammation in the transition from asymptomatic to overt myocardial dysfunction requires further investigation. Chronic low-grade inflammation has been associated with persistent myocardial injury, fibrosis, and arrhythmogenic substrate formation. Late gadolinium enhancement (LGE) patterns observed in cardiac magnetic resonance imaging (MRI) studies of patients with ICI-induced myocarditis may indicate residual fibrotic remodeling, raising concerns about long-term cardiac complications beyond the acute phase. A deeper understanding of these mechanisms is essential for developing surveillance protocols that enable early detection and timely intervention in patients receiving ICIs [6].

This case series provides an in-depth examination of four cases of late-onset immune myocarditis associated with ICI therapy and its management. Additionally, it presents a comprehensive literature review on the subject.

## 2. Case Reports

### 2.1. Case 1

A 56-year-old male patient diagnosed with clear cell renal cell carcinoma was initiated on pembrolizumab and axitinib therapy following disease progression on pazopanib. His medical history included atrial fibrillation, diabetes mellitus, and hypertension.

Six months after receiving the first dose of combination therapy, the patient developed symptoms of chest tightness and shortness of breath. The physical examination was unremarkable. Electrocardiography (ECG) showed sinus tachycardia with a heart rate of 150 bpm and ST-segment depression in leads V4, V5, and V6. Echocardiography revealed a left ventricular ejection fraction (LVEF) of 25% (normal >50%), a dilated left ventricle, and biatrial dilatation. The cardiac troponin T (cTnT) level was 7.4 ng/mL (normal <0.014), and the N-terminal pro-brain natriuretic peptide (NT-proBNP) level was 2302 ng/L (normal <125).

Coronary angiography showed mild, non-obstructive coronary artery disease, with a 20% stenosis in the left anterior descending artery (LAD). However, this finding was deemed clinically insignificant, and no intervention was required. Based on the patient’s clinical presentation, biomarker elevation, and characteristic cardiac MRI findings, a diagnosis of ICI-related myocarditis was established. Cardiac MRI was performed to confirm the diagnosis.

Cardiac MRI demonstrated edema and inflammation consistent with acute myocarditis, predominantly affecting the basal, septal, and inferior segments. The LVEF remained at 25%, with a dilated left ventricle and biatrial enlargement. Secondary causes, including viral and autoimmune markers, were negative.

Based on the clinical symptoms, elevated myocardial injury biomarkers, ECG and echocardiographic abnormalities, and characteristic findings on the cardiac MRI, ICI-related myocarditis was diagnosed. The condition was classified as grade 3 according to the Common Terminology Criteria for Adverse Events (CTCAE) version 5.0. The patient also developed grade 4 immune-related hepatitis. The liver enzyme levels were significantly elevated, with AST at 200 U/L (normal range 0–30 U/L) and ALT at 240 U/L (normal range 0–40 U/L), while the cholestatic markers and bilirubin levels remained within normal limits. According to CTCAE version 5.0, this was classified as a grade 3 adverse event. The condition was successfully managed with corticosteroid therapy.

Recent reports suggest that ICI therapy can trigger multi-organ irAEs, including myocarditis and hepatitis, through shared immunopathological mechanisms. The simultaneous occurrence of myocarditis and immune-related hepatitis may be attributed to CD4+ T cell-mediated inflammation and pro-inflammatory cytokine activation, including TNF-α (tumour necrosis factor-alfa), granzyme B, and interferon-gamma, which play a pivotal role in ICI-induced immune toxicity [7].

Additionally, cross-reactivity between cardiac and hepatic antigens has been proposed as a potential mechanism underlying simultaneous organ involvement in irAEs [7]. A case series reported instances of concurrent myocarditis, myositis, and myasthenia gravis in ICI-treated patients, highlighting the systemic nature of immune activation induced by checkpoint inhibitors [8]. This suggests that myocarditis and hepatitis may not be independent events but rather manifestations of a broader immune dysregulation syndrome affecting multiple organ systems.

Given the high morbidity and mortality associated with ICI-induced myocarditis, particularly when occurring alongside other immune-mediated toxicities, early recognition and aggressive immunosuppressive management are critical for improving patient outcomes [8]. Further research is needed to elucidate the precise molecular pathways linking cardiac and hepatic immune toxicities in patients receiving ICI therapy.

Immunotherapy was permanently discontinued. The patient was treated with methylprednisolone at a dose of 1 mg/kg. Due to the absence of a clinical response and persistent cardiac biomarker elevation by the third day, intravenous immunoglobulin (IVIG) therapy was initiated at a dose of 1 g/kg for five days, while methylprednisolone was continued concurrently. Methylprednisolone was gradually tapered off after the fourth week. A reduction in cardiac biomarkers was observed. Corticosteroid therapy was discontinued at the three-month mark, and follow-up cardiac MRI demonstrated an improvement in the LVEF to 47%. The left ventricle exhibited hypokinetic segments. Additionally, findings suggestive of inflammation and potential fibrosis were observed in the basal inferoseptal and anteroseptal walls, as well as in the mid-inferior segments of the left ventricle. Compared to the previous examination, there was significant improvement. Seven months after the diagnosis of myocarditis, the patient succumbed to the disease progression.

### 2.2. Case 2

A 78-year-old female patient diagnosed with clear cell renal cell carcinoma was initiated on nivolumab therapy following disease progression, despite prior treatment with interferon-alpha and pazopanib. Her medical history included atrial fibrillation and hypertension.

During the 12th cycle of nivolumab therapy (corresponding to the sixth month of treatment), the patient developed symptoms of bradycardia and weakness. The physical examination yielded no noteworthy findings. ECG demonstrated sinus bradycardia with a heart rate of 40 beats per minute (bpm) and ST-segment depression in leads D1, aVL, V4, V5, and V6. Echocardiography revealed a LVEF of 15%, moderate aortic regurgitation, and a dilated left ventricle. The cTnT level was 500 ng/mL, and NT-proBNP was 30,000 ng/L. Coronary angiography demonstrated a 15% stenosis in the right coronary artery (RCA), which was considered hemodynamically insignificant. Given the absence of obstructive coronary artery disease, the diagnosis of ICI-related myocarditis was confirmed based on biomarker elevation and the cardiac MRI findings.

Cardiac MRI revealed widespread myocardial edema, moderate aortic regurgitation, and severe impairment of the left ventricular function. When evaluated in conjunction with the patient’s clinical, radiological, and laboratory findings, these results were consistent with a diagnosis of myocarditis. Secondary causes, including viral and autoimmune markers, were ruled out.

ICI-related myocarditis was diagnosed based on the patient’s clinical symptoms, elevated myocardial injury biomarkers, ECG and echocardiographic abnormalities, and the characteristic cardiac MRI findings. The condition was classified as grade 4 according to CTCAE version 5.0.

Immunotherapy was permanently discontinued. The patient was treated with methylprednisolone at a dose of 1 mg/kg. A decrease in troponin levels was observed with corticosteroid therapy. However, within the first month of treatment, the patient was admitted to the intensive care unit due to cardiogenic shock and died shortly thereafter.

### 2.3. Case 3

A 64-year-old male patient diagnosed with nasopharyngeal squamous cell carcinoma was initiated on nivolumab therapy after progression on carboplatin and gemcitabine. There was no known prior cardiac or non-cardiac comorbidity.

During the 36th cycle of nivolumab therapy (20th month of treatment), the patient developed symptoms of bradycardia, weakness, and hypotension. The physical examination yielded no noteworthy findings. An ECG revealed the presence of widespread ventricular extrasystoles (VES) and sinus bradycardia. Twenty-four-hour Holter monitoring recorded a total of 5000 VES and an average heart rate of 45 beats per minute. No instances of ventricular tachycardia or ventricular fibrillation were observed, nor were there any pauses exceeding three seconds. Echocardiography demonstrated a LVEF of 60% and moderate aortic regurgitation. The level of cTnT was 0.5 ng/mL, while the level of NT-proBNP was 1200 ng/L. Secondary causes of hypotension, including infection and pituitary or adrenal disorders, were excluded. Coronary angiography showed a 30% stenosis in the circumflex artery (CX), which was not clinically significant. The patient’s symptoms, elevated cardiac biomarkers, and cardiac MRI findings supported a diagnosis of ICI-related myocarditis. A cardiac MRI was scheduled.

**Figure 1:**
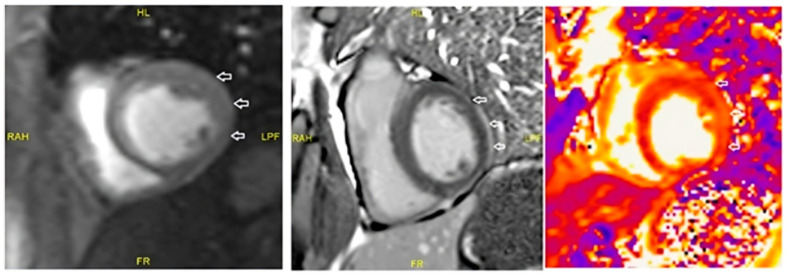
**Case 3**. Cardiac MRI revealed subepicardial linear contrast enhancement in the lateral wall of the left bentricle (indiacated by an arrow). When evaluated with T2 mapping images, the findings were predominately consistent with a diagnosis of myocarditis, particularly considering the clinical findings. Moderate aortic regurgitation was observed. Secondary casuses, including viral and autoimmune markers, were ruled out.

Cardiac MRI findings of Case 3 are shown in Figure 1.

A diagnosis of ICI-related myocarditis was made based on the patient’s clinical symptoms, elevated myocardial injury biomarkers, ECG and echocardiographic changes, and characteristic findings on the cardiac MRI. The diagnosis was graded as grade 4 according to CTCAE version 5.0.

Immunotherapy was permanently discontinued. The patient was treated with methylprednisolone 1000 mg (pulse steroid) for a period of three days, followed by a dose of 1 mg/kg/day. Due to persistent hypotension, noradrenaline was introduced as an inotropic agent for hemodynamic support. Despite inotropic support, the patient’s bradycardia and hypotension remained unimproved, thus prompting the initiation of mycophenolate mofetil treatment at a dosage of 2 × 1 g, while corticosteroid therapy was maintained. In the second week of combined immunosuppressive therapy, a reduction in the cardiac biomarkers and an improvement in hypotension and bradycardia were observed. Mycophenolate therapy was terminated after one month, and corticosteroid therapy was gradually discontinued and ultimately terminated after two months. A cardiac MRI performed in the second month of treatment showed normal findings. The abnormalities observed in the initial MRI, which were indicative of myocarditis, were no longer present.

In this case, mycophenolate mofetil (MMF) was chosen as a second-line immunosuppressive agent due to the patient’s refractory myocarditis despite corticosteroid therapy. According to current recommendations, patients with steroid-refractory irAEs, including myocarditis, require additional immunosuppressive therapy to control inflammation and prevent further cardiac damage [9].

MMF selectively inhibits inosine monophosphate dehydrogenase, thereby suppressing T- and B-cell proliferation, which plays a critical role in ICI-induced myocarditis [7]. Clinical guidelines suggest MMF as a preferred option when corticosteroid therapy alone is insufficient, as it has demonstrated efficacy in controlling severe and persistent irAEs while reducing long-term steroid-associated complications [10].

Recent studies have highlighted MMF’s effectiveness in mitigating cardiac inflammation and preventing disease progression in steroid-refractory myocarditis. Its immunosuppressive effects contribute to limiting myocardial fibrosis and restoring cardiac function, which are crucial in managing immune-related myocarditis. Furthermore, retrospective analyses and an expert consensus recommend MMF in cases where prolonged steroid use poses significant risks, such as hyperglycemia, osteoporosis, and secondary infections. The ability of MMF to offer sustained immunosuppression with a favorable safety profile makes it a viable option in such clinical scenarios [9].

As clinical guidelines do not provide a definitive recommendation for all cases, the decision to initiate MMF was individualized based on the patient’s incomplete response to corticosteroids and the need to mitigate potential steroid-related adverse effects. Following MMF initiation, a gradual improvement in cardiac function and biomarker stabilization was observed. Further studies are warranted to optimize the management of steroid-refractory ICI myocarditis [11].

The patient succumbed to the disease six months after the diagnosis of myocarditis.

### 2.4. Case 4

A 74-year-old male patient diagnosed with lung squamous cell carcinoma was initiated on first-line treatment with carboplatin, paclitaxel, and pembrolizumab. He had a known history of diabetes mellitus and hypertension.

During the 11th cycle of nivolumab therapy (6th month of treatment), the patient developed symptoms of tachycardia, weakness, and shortness of breath. The physical examination yielded no noteworthy findings. An ECG demonstrated sinus tachycardia and a new onset of VES. Twenty-four-hour Holter monitoring revealed a total of 2000 ventricular extrasystoles and an average heart rate of 130 beats per minute. No instances of ventricular tachycardia, ventricular fibrillation, or supraventricular tachycardia were observed. Echocardiography demonstrated a LVEF of 60%. The cTnT level was 0.001 ng/mL. Similarly, the level of NT-proBNP was 800 ng/L. Coronary angiography demonstrated a mild, non-obstructive 40% stenosis in the CX, which was considered hemodynamically insignificant. Given the absence of significant coronary artery disease, the diagnosis of ICI-related myocarditis was confirmed based on biomarker elevation and the cardiac MRI findings. Cardiac MRI revealed late subepicardial linear contrast enhancement in the lateral wall of the basal segment of the left ventricle. Secondary etiologies, including viral markers and autoimmune markers, were excluded.

ICI-related myocarditis was diagnosed based on the clinical symptoms, elevated myocardial injury biomarkers, ECG and echocardiographic changes, and characteristic findings on the cardiac MRI. This was classified as grade 2 according to CTCAE version 5.0. Prior to the onset of immune myocarditis, the patient had also developed grade 2 immune pneumonitis and grade 1 immune thyroiditis. Immunotherapy was initiated after the immune-related pneumonitis was reduced to grade 1 with methylprednisolone and thyroiditis was controlled with levothyroxine replacement, immunotherapy was started.

Immunotherapy was permanently discontinued. The patient administered methylprednisolone at a dosage of 1 mg/kg. The patient exhibited an improvement in tachycardia from the third day of the methylprednisolone administration. Methylprednisolone was gradually discontinued after the fourth week. A reduction in the cardiac biomarkers was noted. Corticosteroid therapy was discontinued after a two-month period, and a control cardiac MRI demonstrated stable T1 mapping values from the interventricular septum and persistent late contrast enhancement in the inferolateral wall.

Late gadolinium enhancement observed in cardiac MRI serves as a key marker of myocardial fibrosis and plays a crucial role in the long-term prognosis of myocarditis patients. Studies have demonstrated that the presence of LGE is associated with an increased risk of heart failure, arrhythmias, and adverse cardiac events, even after the acute phase of myocarditis has resolved [12]. The persistence of LGE suggests ongoing fibrotic remodeling, which may contribute to ventricular dysfunction and long-term cardiac complications [13].

Given these findings, regular follow-ups with cardiac MRI are strongly recommended to assess myocardial recovery and detect potential late complications. The literature suggests that a follow-up MRI at 3 to 6 months post-myocarditis can provide critical insights into disease progression and guide the long-term management. Close monitoring remains essential, particularly in patients with persistent LGE, as they may require further risk stratification and additional cardiac surveillance [12,13].

No significant progress was observed. The patient is currently under observation without the use of any medication and is undergoing active monitoring for any potential cancer developments. A summary of the cases is shown in Table 1 below.

## 3. Discussion

Although ICIs have revolutionized cancer therapy, their associated hyperactivation of the immune system can trigger autoimmune disorders, including fulminant ICI myocarditis. While rare, immune-related myocarditis carries a high mortality rate, estimated at 40–50% [14,15]. Its prevalence is approximately 1.14% among patients receiving ICIs but this number is rising due to increased clinical awareness [16,17].

ICI therapy-related myocarditis typically manifests during the early stages of treatment, most often within the first 90 days, with a median onset time of approximately 34 days, usually occurring within the first three infusions. However, late-onset cases have also been reported, with the latest known case occurring approximately 500 days after treatment initiation. Increasing evidence highlights the potential for delayed irAEs even after discontinuation of ICI therapy, particularly in cardiac manifestations, which are associated with an increased incidence of left ventricular systolic dysfunction and heart failure [8,14,15].

Several hypotheses have been suggested to explain the pathophysiology of ICI myocarditis. One hypothesis posits that T cells may be targeting cardiac-specific antigens or antigens shared between cardiac tissue and tumors.

The onset of ICI-induced myocarditis varies, with early cases (within 21 days) driven by acute T-cell activation, while late-onset myocarditis can manifest months to years later [7]. The delayed onset is hypothesized to result from gradual autoimmune activation, sustained cytokine release, or prolonged immune system stimulation [8]. Persistent antigen presentation, along with prolonged T-cell and macrophage activation, may contribute to chronic low-grade myocardial inflammation that eventually progresses to overt myocarditis [18]. A case report described myocarditis presenting 304 days after ICI therapy initiation, highlighting the potential for late immune-mediated cardiac damage [7].

Additionally, a genetic predisposition and pre-existing subclinical myocardial inflammation may contribute to the timing of disease presentation. Some patients may harbor underlying immune dysregulation that predisposes them to delayed inflammatory responses, while others may experience immune tolerance breakdown over time [10].

Subclinical myocarditis represents an early phase of immune-mediated myocardial injury, in which patients may exhibit elevated cardiac biomarkers or subtle functional impairments detectable through imaging [13]. Over time, chronic inflammation promotes myocardial fibrosis, leading to ventricular dysfunction and increased arrhythmogenic risk [6]. A case series reported that patients with subclinical troponin elevation may precede symptomatic myocarditis, highlighting the need for early detection [17]. This underscores the importance of early detection through serial cardiac biomarker assessment and imaging modalities such as late gadolinium enhancement on MRI, which has been shown to predict long-term cardiac dysfunction [15].

Given these findings, patients receiving ICI therapy should undergo routine cardiac surveillance, especially those with persistently elevated biomarkers or abnormal imaging findings, even in the absence of symptoms [19].

Corticosteroids are the mainstay treatment for ICI-induced myocarditis; however, their efficacy may differ between early- and late-onset disease [20]. In early-onset myocarditis, where acute T-cell-mediated inflammation dominates, high-dose corticosteroids are often effective in suppressing inflammation and restoring myocardial function [1]. In contrast, late-onset myocarditis is often characterized by myocardial fibrosis, a process less responsive to corticosteroid therapy [21]. Studies suggest that fibrotic myocardial remodeling may persist despite aggressive immunosuppressive therapy, leading to ongoing ventricular dysfunction. Given this, alternative therapeutic strategies, such as antifibrotic agents or T-cell targeted therapies, may be necessary in steroid-resistant cases [10]. Further research is needed to define the optimal immunosuppressive regimen for late-onset myocarditis.

The existence of shared antigens has been demonstrated through the sequencing of T-cell receptor CDR3 regions, which revealed similarities between tumor and cardiac/skeletal muscle sequences. While further mechanistic studies are required, it is plausible that neoantigens, which enhance tumor immunogenicity, may also cross-react with the myocardium, thereby stimulating a profound autoimmune response. The continuous creation of neoantigens due to defective DNA repair may sustain prolonged T-cell stimulation, potentially leading to late-onset autoimmunity. A comprehensive understanding of the mechanisms underlying ICI therapy-related myocarditis is of paramount importance for the accurate long-term risk stratification of patients and the development of effective therapeutic strategies. In patients who achieve tumor control with immunotherapy, the recognition of late-onset ICI therapy-related myocarditis is of paramount importance. Furthermore, the role of extended vigilance for irAEs requires further investigation [22,23].

Recent studies have highlighted the emergence of subclinical myocarditis as a significant concern following ICI therapy. This condition often presents without overt symptoms, making early detection challenging. The pathophysiology of ICI-associated myocarditis shares similarities with chronic viral myocarditis, particularly in terms of immune-mediated mechanisms.

In a case reported by Zhang et al., a 60-year-old male undergoing ICI treatment for lung cancer exhibited elevated cardiac biomarkers without noticeable symptoms. Despite the absence of clinical manifestations, diagnostic evaluations confirmed myocarditis. The patient responded well to high-dose corticosteroid therapy, underscoring the importance of early recognition and intervention in such subclinical cases [24].

A comprehensive review by Mir et al. analyzed 244 cases of ICI-associated myocarditis. The study found that 10.1% of these cases were asymptomatic, with myocarditis detected through routine monitoring of cardiac biomarkers and electrocardiograms. This finding emphasizes the necessity for vigilant cardiac monitoring in patients receiving ICI therapy to identify subclinical presentations promptly [7].

The underlying mechanisms of ICI-related myocarditis involve immune system dysregulation, leading to T-cell infiltration of the myocardium. This mirrors the pathogenesis observed in viral myocarditis, where an initial infection triggers an autoimmune response against cardiac tissues. Such immune responses can result in myocardial inflammation and fibrosis, potentially progressing to dilated cardiomyopathy and ventricular arrhythmias if not addressed in a timely manner [25].

Given the potential severity of myocarditis, even in its subclinical form, it is crucial for clinicians to maintain a high index of suspicion when managing patients on ICI therapy. Regular monitoring of cardiac biomarkers, electrocardiographic assessments, and the consideration of early intervention strategies are essential to mitigate the risks associated with this emerging adverse event.

A diagnosis of immune myocarditis was made in three of our patients at the 6-month follow-up after treatment, and in one patient at the 20-month follow-up. What was most striking was that all four cases occurred late.

Studies indicate that patients with comorbidities like diabetes, hypertension, and cardiac disease have a higher risk of immune-mediated myocarditis. Moreover, the risk associated with combination immunotherapies has been found to be greater than that of monotherapy [15]. Our case series did not involve the use of combination immunotherapy. Nevertheless, three patients exhibited one or more comorbidities, namely diabetes, hypertension, and cardiac disease. Considering the elevated risk of immune-mediated myocarditis in patients with severe comorbidities undergoing combination immunotherapy, close cardiac monitoring is crucial.

ICI therapy-induced myocarditis is a serious adverse event that can present with a wide range of clinical manifestations. Common symptoms include shortness of breath, hypotension, palpitations, chest pain, heart failure, fatigue, weakness, and myalgia. This delayed presentation may reflect ongoing inflammation and cardiac remodelling. The predominant presenting symptoms are shortness of breath (prevalence: 55–68%), palpitations (5–33%), and chest pain (13–28%). A higher incidence of LV systolic dysfunction (37–74%) is observed in late-onset cases, which may account for the increased prevalence of heart failure symptoms. Patients may also experience non-specific symptoms such as fatigue or weakness [14,15,26]. Myocarditis should be considered as a differential diagnosis, even in cases with non-specific symptoms. Moreover, it may be incidentally detected during routine troponin surveillance [8]. In our patients, cardiac troponin levels were elevated in three patients, while it was within the normal range in one patient.

The Society of Immunotherapy in Cancer (SITC), the European Society of Medical Oncology (ESMO), and the recent European Society of Cardiology (ESC) guidelines recommend obtaining baseline basic cardiac biomarkers such as troponin I, BNP, and ECG prior to the start of ICI therapy to establish an accurate reference for comparison, whereas the American Society of Clinical Oncology (ASCO) guidelines do not, stating that “there is no clear evidence regarding the efficacy or value of routine ECGs or troponin measurements in patients receiving checkpoint inhibitor therapy” [27,28,29].

ICI-induced myocarditis can be challenging to diagnose due to the absence of specific biomarkers, which can prolong the diagnostic process for up to 90 days. A combination of clinical, laboratory, and imaging tests is often necessary for an accurate diagnosis.

An endomyocardial biopsy (EMB) is considered the gold standard for the definitive diagnosis of immune-mediated myocarditis, as it provides histopathological evidence of myocardial inflammation, necrosis, and fibrosis. However, due to its invasive nature, procedural risks, and the potential sampling error, EMB is not always performed in clinical practice [30]. The ESC and SITC guidelines acknowledge that, while EMB can confirm myocarditis, non-invasive imaging modalities, particularly cardiac MRI, have become essential diagnostic tools for the assessment of myocarditis [25].

Cardiac biomarkers like troponin and BNP can be elevated but are not always specific. ECG may show abnormalities, while echocardiography can reveal a reduced left ventricular ejection fraction. Cardiac MRI is considered the gold standard for non-invasive diagnosis, with LGE (late gadolinium enhancement) and T1/T2 mapping providing valuable information. PET has a limited role, but 68Ga-DOTATOC PET shows promise. An endomyocardial biopsy is invasive and may be considered in severe cases or when non-invasive tests are inconclusive.

The ESC guidelines recommend a combination of clinical, laboratory, and imaging findings for diagnosis, and the Bonaca criteria can be used to assess the probability of ICI myocarditis [25,31,32].

In our case series, an EMB was not performed due to its invasive nature. Since all patients had a clear history of ICI therapy, along with clinical and laboratory findings strongly indicative of myocarditis, after ruling out other potential causes such as viral, autoimmune, and toxic myocarditis, a biopsy was deemed unnecessary. Current guidelines suggest that while an EMB can provide definitive histopathological confirmation, ICI-related myocarditis can often be reliably diagnosed using a combination of clinical presentation, biomarker elevation, ECG changes, echocardiography, and characteristic cardiac MRI findings. Moreover, EMB findings may not necessarily alter the clinical management, as treatment decisions are typically based on non-invasive diagnostic criteria [19]. Further studies are needed to refine the role of EMB in ICI-induced myocarditis and determine its impact on treatment strategies.

The initial step in managing suspected ICI therapy-induced myocarditis is to immediately halt ICI therapy while conducting diagnostic tests. This approach is endorsed by all the major medical societies [11].

Corticosteroids are the first-line treatment for irAEs, including myocarditis. Guidelines from the European Society of Cardiology (ESC) and the European Society for Medical Oncology (ESMO) recommend initiating high-dose corticosteroids (e.g., methylprednisolone) for the first few days, followed by a gradual reduction in dosage. The Society for Immunotherapy of Cancer (SITC) and the American Society of Clinical Oncology (ASCO) also recommend high-dose corticosteroids for confirmed or highly suspected myocarditis.

In cases where corticosteroids are insufficient, additional therapies may be considered, including IVIG, tocilizumab, alemtuzumab, anti-thymocyte immunoglobulin, mycophenolate mofetil, and abatacept. Recent studies have shown promising results with a combination therapy involving low-dose steroids, abatacept, and ruxolitinib in severe cases of ICIs-induced myocarditis [10,28,29].

Our cases illustrate the significance of recognizing that ICIs-related myocarditis can manifest as a delayed irAE. These cases underscore the importance of awareness, developing early clinical suspicion, and the rapid detection of delayed-onset ICI-induced myocarditis to prevent decompensation and the development of fulminant myocarditis. As the indications for immunotherapy continue to expand, the ability to recognize delayed-onset ICI-related myocarditis will become increasingly crucial as the life expectancy after cancer treatment continues to improve.

In all cases methylprednisolone was administered, while one patient received IVIG, and another received mycophenolate mofetil in addition. Three patients were treated with methylprednisolone at a dosage of 1 mg/kg. In one patient, the initial dose was 1000 mg of methylprednisolone for three days, followed by a maintenance dose of 1 mg/kg. The high-dose methylprednisolone regimen was continued for a minimum of one month and was tapered based on the patient’s response. Mycophenolate mofetil was initiated at a dose of 2 × 1000 mg for one month in a patient who did not respond to three days of methylprednisolone. IVIG was administered at a dose of 5 g/kg for five days in one patient. Considering the patient’s rapidly deteriorating cardiac condition and the established role of intravenous immunoglobulin (IVIG) in the management of similar cases, we opted for immediate IVIG administration. The patient exhibited a prompt clinical improvement following this intervention.

The occurrence of immune myocarditis-related death was observed in only one of the four patients who developed ICI-induced myocarditis. Two patients died because of disease progression and one patient remains alive. To the best of our knowledge, the latest case of immune myocarditis was previously reported to have developed on the 500th day. One of our cases developed immune myocarditis on the 600th day after the initiation of immunotherapy, which we believe to be the latest case reported in the literature.

## 4. Conclusions

ICI therapy has markedly enhanced the efficacy of cancer treatment. However, it is associated with a range of irAEs, including myocarditis. Although early-onset ICI-related myocarditis is well documented, recent reports of late-onset cases emphasize the necessity for long-term surveillance.

Our case series highlights the importance of increased awareness of late-onset ICI-related myocarditis, which can present with a wide range of clinical manifestations. The early diagnosis and prompt initiation of immunosuppressive therapy are paramount for improving patient outcomes.

Previous studies have consistently demonstrated that cardiotoxicity associated with checkpoint inhibitors typically presents early, shortly after infusion [22,32,33,34,35]. However, our cohort of patients with immune myocarditis exhibited a late-onset phenotype, deviating from the established literature [3,36].

As the use of ICI therapy continues to expand, recognizing the risk of late-onset ICI-induced myocarditis and implementing strategies for its early detection and intervention are imperative. Doing so will enhance both the safety and efficacy of this transformative cancer treatment.

## Figures and Tables

**Table 1:** Summary of our cases.

The Levels of Troponin and NT-proBNP Presented in the Table Correspond to the Peak Values Recorded at the Time of Diagnosis, Providing an Accurate Reflection of the Severity of Myocardial Injury.				
	Case-1	Case-2	Case-3	Case-4
ICI-agent	Pembrolizumab	Nivolumab	Nivolumab	Pembrolizumab
Primary diagnosis	RCC	RCC	Nasopharynx	Lung
ICIs to onset of myocarditis (months)	6	6	20	6
Age	56	78	64	74
Sex	male	male	male	male
CV risk factors	AF, DM, HT	AF, HT	none	DM, HT
Myocarditis presentation	chest tightness and shortness of breath	bradycardia and weakness	bradycardia, weakness, and hypotension	tachycardia, weakness, and shortness of breath
cTnT(ng/mL)	7.4	500	0.5	0.001
NT-proBNP (ng/L)	2302	30,000	1200	800
ECG	sinus tachycardia, ST depression in leads V4, V5, and V6	sinus bradycardia, ST depression in leads D1, aVL, V4, V5, and V6	ventricular extrasystoles and sinus bradycardia	sinus tachycardia and new onset of ventricular extrasystoles
ECO	LVEF: 25%, dilated left ventricle, and biatrial dilatation	LVEF: 15%, moderate aortic regurgitation, and a dilated left ventricle	LVEF of 60% and moderate aortic regurgitation	LVEF of 60%
cMRI	diffuse	diffuse	subepicardial	subepicardial
Metylprednizolon, dose mg/kg) and time (months)	1 mg/kg, 3 months	1 mg/kg, 3 weeks	1000 mg 3 days and 1 mg/kg 2 months	1 mg/kg, 2 months
Other IS-agents	IVIG	none	mycophenolate mofetil	none
Causes of death	disease progress	cardiogenic shock	disease progress	alive
ICIs: Immune checkpoint inhibitors CV: Cardiovascular cTnT: Cardiac troponin T NT-proBNP: N-terminal pro-brain natriuretic peptide ECG: Electrocardiogram ECO: Echocardiography cMRI: Cardiac magnetic resonance imaging IS: Immunosuppressive			HT: Hypertension DM: Diabetes mellitus AF: Atrial fibrillation IVIG: Intravenous immunoglobulin RCC: Renal cell carcinoma LVEF: Left ventricular ejection fraction	

## Data Availability

Not applicable.
